# A Chinese Cross-Sectional Study on Symptoms in Aging Males: Prevalence and Associated Factors

**DOI:** 10.1177/1557988319838113

**Published:** 2019-03-12

**Authors:** Hongjun Li, Xinyu Zhang, Haibo Wang, Bin Yang, Ni Li, Zhigang Ji

**Affiliations:** 1 Department of Urology, Peking Union Medical College Hospital, Chinese Academy of Medical Sciences and Peking Union Medical College, Beijing, China; 2 Department of Urology, Navy General Hospital of the Chinese People’s Liberation Army, Beijing, China; 3 Clinical Research Institute, Peking University, Beijing, China; 4 Program Office for Cancer Screening in Urban China, Cancer Hospital and Institute, Chinese Academy of Medical Sciences, Beijing, China

**Keywords:** development and aging, social determinants of health, psychosocial and cultural issues, health-related quality of life, general health and wellness

## Abstract

The Aging Male Symptoms (AMS) scale is a questionnaire designed for assessing health-related quality of life and aging-related symptoms in men. Additional knowledge of the severity of aging symptoms in males revealed by high AMS scores and the factors associated with it in the Chinese population is required. A nationally representative prevalence and risk factor estimate of AMS scores was performed to identify the associated factors for AMS severity in China. Men aged between 35 and 70 years were recruited at 33 study centers in 21 provinces, 4 municipalities, and 4 autonomous regions. The prevalence of high AMS scores and its association with demographic, anatomical, lifestyle, and clinical variables were evaluated. Chi-square tests and logistic regression models were used for analysis. Odds ratios (ORs) and 95% confidence intervals were calculated. In this study, 918 of 9,164 (10%, *p* < .001) men aged between 35 and 70 years, had AMS scores ≥50. Univariate and multivariable analyses showed that an age of >40 years, poor marital relations, type 2 diabetes mellitus (T2DM), history of fracture, and smoking ≥25 cigarettes per day were the major factors that were associated with the severity of AMS (OR ≥2; *p* < .05). Hypertension, low income, a low education level, alcohol consumption, lack of exercise, and a waist-to-hip ratio ≥0.9 were also moderately associated with AMS severity (OR 1–2; *p* < .05). The current study revealed the nationally representative prevalence of severe AMS scores in Chinese men and the factors associated with severe AMS. Antiaging intervention studies should target men with specific associated factors.

Healthy aging is an indicator of the health status of any society (Sander et al., 2015). The improvement in medical facilities has led to a demographic shift favoring the older adult population, making aging-related quality of life (QoL) significant in many countries (Netuveli & Blane, 2008). The symptoms associated with aging in females are more obvious and are well documented, whereas, in the case of males, the symptoms are subtle and, more often, they are not recognized (Abram McBride, Carson, & Coward, 2016). The symptoms associated with the physiological changes of aging most often overlap with disease-mediated pathological symptoms, which require specific diagnosis (McGill, Shoskes, & Sabanegh, 2012). The different symptoms of aging in males such as fatigue, weakness, depression, decreased libido, and insomnia affect the health-related quality of life (HRQoL) in aging males and are categorized into those that affect the mind (psychological), body (somato-vegetative), and sexual ability (Jankowska, Szklarska, Lopuszanska, & Medras, 2008). The functional limitations and increased health-care burden caused by the different symptoms of aging have significant economic consequences (Netuveli & Blane, 2008). Because of population growth and aging, it has been estimated that the global burden of depression has increased by 37.5% between 1990 and 2010 (Ferrari et al., 2013); clinical consequences of osteoporosis are also associated with increased economic burden (Maggi, Schulman, Quinton, Langham, & Uhl-Hochgraeber, 2007; Watts et al., 2012).

The Aging Male Symptoms (AMS) scale analyzes the three parameters of aging and it grades the quality of life in aging males (Heinemann et al., 2003). Many factors have been known to be associated with HRQoL, which varies among countries. In a previous cross-sectional study in Hong Kong, smoking, drinking, self-perceived obesity, and self-reported metabolic syndrome were significantly associated with aging. The same study reported the severity of AMS to be significantly associated with age (Yuen, Ng, Chiu, Teoh, & Yee, 2016). However, the direct association of various demographic and clinical variables with the severity of AMS was not analyzed. Although multiple studies had used the AMS scale to assess HRQoL, the studies were done within geographically confined urban locations in China with limited number of participants and so may not reflect the precise HRQoL among Chinese men (W. Chen et al., 2013; Chueh et al., 2012; Li et al., 2016; Sun et al., 2012). A national survey of the prevalence and severity of AMS in China has not been reported. The associated predisposing factors that favor more severe AMS scales have also not been elucidated. Due to an imbalance in economic development between urban and rural regions in China, the aging population in rural China has increased in the past decade. It was hypothesized that a nationally representative AMS survey will provide a more accurate analysis of the lifestyle, socioeconomic, and health-related factors that are associated with AMS severity (AMS score ≥50) in Chinese men.

Therefore, a nationally representative prevalence and risk factor estimate for aging male symptoms utilizing the AMS questionnaire was carried out to evaluate the associated factors favoring high AMS scores in Chinese men.

## Materials and Methods

### Study Design and Participants

A cross-sectional nationally representative prevalence and risk factor estimation study was performed in 33 centers from 21 provinces, 4 municipalities, and 4 autonomous regions located across mainland China between January 2010 and October 2014. The study included community males attending a public lecture on men’s health, between 35 and 70 years of age who were briefed about the purpose and the scope of the study before enrolling.

At the beginning of the study, one or two hospitals in each region were chosen and expert physicians from Tier 3 hospitals were asked to assist in the supervision of the survey in community areas as a pilot study followed by community-wise research. The head of each local community committee was invited to recruit a cohort of men aged between 35 and 70 years who subsequently volunteered to receive health education counseling in the community. Expert physicians explained to the volunteers about climacteric health, androgens and health, prostate disease, and so forth, for an hour and guided them in filling out the questionnaires before starting the survey. Completion of the questionnaire was facilitated by professional doctors and nurses to avoid illogical mistakes in the process of filling out the questionnaire. Finally, a total of 10,000 questionnaires were collected as the cutoff number from the 33 centers, and each center planned to collect 300–350 samples. A total of 836 (8.36%) questionnaires with incomplete data were not included for the analysis. A total of 9,164 questionnaires with complete information were gathered in this study.

The following criteria were used to exclude men with (a) a requirement of assisted living; (b) endocrine disorders affecting sex hormone levels; (c) a history of psychotic or cognitive disorders; (d) debilitating diseases such as cancer and heart disease; and (e) a history of pelvic surgery; in addition, patients who were on hormonal or psychotropic agents were excluded. Further, physical and clinical examinations were carried out to exclude men with epididymitis, urinary tract infection, venereal disease, severe Peyronie’s disease, phimosis, and paraphimosis, which may have confounded the study results. A signed, written informed consent was obtained from all participants prior to participation. The study was approved by the medical ethics committee of each institution (No. S-214) and was performed in accordance with the Declaration of Helsinki with regard to the ethical treatment of human subjects.

### Study Locations

This study was a nationally representative prevalence and risk factor estimate for high AMS scores and the data were independently collected by cluster sampling with geographical distribution from 33 study centers including Gansu, Sichuan, Guangxi, Gansu, Hainan, Heilongjiang, Hebei, Henan, Jiangsu, Jiangsu, Jilin, Anhui, Inner Mongolia, Shanxi, Shanxi, Shandong, Guangzhou, Beijing, Jiangxi, Liaoning, Hubei, Hebei, Shaanxi, Ningxia, Xinjiang, Hunan, Chongqing, Shanghai, Tianjin, and Beijing. The multicenter stage samples were then combined for further statistical analyses. Hong Kong, Taiwan, Macau, Tibet, and Qinghai were not represented in the analysis.

### Study Variables

The demographic variables considered for the study were age, ethnicity, education level, income, and marital status. Lifestyle variables included exercising routinely, smoking, alcohol consumption, and satisfaction with marital relations. Both demographic and lifestyle variables were answered by the participant through a questionnaire. Anatomical variables data including body mass index (BMI) and waist-to-hip ratio (WHR) data were measured by physical examination. The following clinical variables, which were not accounted for in the AMS questionnaire were collected by clinical examination and interview: type 2 diabetes mellitus (T2DM), hypertension, benign prostate disease, history of stroke, and history of fracture. Participants completed the Chinese version of the AMS questionnaire while general practitioners and urologists were available to clarify questionnaire items privately.

### AMS Assessment

The Chinese version of the AMS questionnaire was used in the present study, the reliability and validity of which have been confirmed in a Chinese population (Chen, Wang, Liu, & Lee, 2007; Kong, Guan, Li, Zhou, & Xiong, 2014). The AMS questionnaire is a 17-item polychotomous questionnaire that uses a 5-point scale (*none* = 1, *mild* = 2, *moderate* = 3, *severe* = 4, and *extremely severe* = 5) to assess various aspects of HRQoL in aging men. The 17 items address mental (sleep disorders, depression, anxiety, etc.), physical (arthralgia, physical activity, muscle strength, etc.), and sexual (morning erections, frequency of sexual activity, etc.) aspects. Possible total AMS scores range from 17 to 85, with higher scores indicating greater severity of symptoms and total scores classified as none/little (17–26), mild (27–36), moderate (37–49), or severe (≥50). Based on their AMS scores, participants were divided into the AMS <50 or AMS ≥50 groups for the analysis.

### Statistical Methods

Descriptive statistics were used to describe the different study variables as dictated by the data type. The association of the demographic, anatomic, lifestyle, and clinical variables with the AMS score was evaluated using chi-square analyses. Pearson’s correlation analysis was performed to analyze the linearity of the AMS score (quantitative) and age. The strength of association between the study variables and the AMS score was evaluated using univariate logistic regression by measuring the odds ratio (OR) and 95% confidence interval (CI) for each variable. The level of statistical significance was set at *p* < .05. Stratified analyses of associations between the clinical variables and the AMS score based on age group (≤40 and >40 years) were also performed. Multivariable models were used to evaluate the overall associations following adjustment for potential confounders. The risk factors found to be significant in the univariate analyses (*p* < .20) were included in the final multivariable model. Stepwise selection was used to determine the final model with entry criteria of *p* = .20 and stay criteria of *p* = .05. Missing values were imputed to check the sensitivity of the analyses.

## Results

### Distribution of AMS Scale and Categorization of the Study Variables

The distribution of demographic, anatomical, and lifestyle variables is given in Table 1. The AMS scores increased linearly with age (*p* < .001). AMS scores ≥50 were recorded for 918 (10.0%) of the participants (95% CI [9.41, 10.65]) and reflects the precision of the prevalence in our study; in the AMS ≥50 group, 876 (95.4%) of the men were over 40 years old. To compare the association of the variables with the AMS score, family income (<¥4,000, ¥4,000–¥5,999, and >¥6,000), BMI (<20, 20–25, 25–30, and >30), and WHR (≥0.9 or <0.9) were arbitrarily categorized into different value ranges for robust analysis. The other variables were divided into ordinal categories. For further analysis, the AMS scores were divided into <50 and ≥50.

**Table 1.** Univariate Analysis of Demographic, Anatomical, and Lifestyle Variables

Variable type	Total men (*N* = 9,164)	AMS <50 (*n* = 8,246)	AMS ≥50 (*n* = 918)	OR [95% CI]	*p*
Demographic					
Age (years)					
≤40	2,135 (23.3%)	2,093 (25.4%)	42 (4.6%)	1.0	
>40	7,029 (76.7%)	6,153 (74.6%)	876 (95.4%)	5.14 [3.72, 7.10]	<.001
Ethnicity					
Han	8,575 (93.6%)	7,714 (93.5%)	861 (93.8%)	1.0	
Others	589 (6.4%)	532 (6.5%)	57 (6.2%)	1.26 [0.92, 1.72]	.158
Education level					
Primary	1,127 (12.3%)	926 (11.2%)	201 (21.9%)	1.0	
Secondary	4,724 (51.5%)	4,185 (50.8%)	539 (58.7%)	0.57 [0.47, 0.69]	<.001
Postsecondary	3,313 (36.2%)	3,135 (38.0%)	178 (19.4%)	0.30 [0.24, 0.37]	<.001
Family income (¥/month)					
<4,000	5,674 (61.9%)	4,983 (60.4%)	691 (75.3%)	1.0	
4,000–5,999	2,290 (25.0%)	2,127 (25.8%)	163 (17.8%)	0.55 [0.45, 0.68]	<.001
≥6,000	1,200 (13.1%)	1,136 (13.8%)	64 (7.0%)	0.46 [0.34, 0.62]	<.001
Anatomical					
Body mass index					
<20	389 (4.2%)	347 (4.2%)	42 (4.6%)	1.0	
20–25	5,559 (60.7%)	4,985 (60.4%)	574 (62.5%)	0.80 [0.56, 1.15]	.229
25–30	2,936 (32.0%)	2,659 (32.3%)	277 (30.2%)	0.72 [0.50, 1.05]	.090
≥30	280 (3.1%)	255 (3.1%)	25 (2.7%)	0.79 [0.45, 1.37]	.393
Waist–hip ratio					
<0.9	2,762 (30.1%)	2,572 (31.2%)	190 (20.7%)	1.0	
≥0.9	6,402 (69.9%)	5,674 (68.8%)	728 (79.3%)	1.33 [1.10, 1.62]	.004
Lifestyle					
Tobacco smoking (cigarettes per day)					
Nonsmoker	4,690 (51.2%)	4,272 (51.8%)	418 (45.5%)	1.0	
<25	3,629 (39.6%)	3,306 (40.1%)	323 (35.2%)	1.18 [1.00, 1.40]	.054
≥25	845 (9.2%)	668 (8.1%)	177 (19.3%)	2.95 [2.35, 3.69]	<.001
Alcohol consumption					
No	6,432 (70.2%)	5,893 (71.5%)	539 (58.7%)	1.0	
Yes	2,732 (29.8%)	2,353 (28.5%)	379 (41.3%)	1.74 [1.50, 2.04]	<.001
Marital relations satisfaction					
Average	5,624 (61.4%)	5,212 (63.2%)	412 (44.9%)	1.0	
Below average	2,918 (31.8%)	2,506 (30.4%)	412 (44.9%)	1.73 [1.47, 2.03]	<.001
Poor	99 (1.1%)	71 (0.9%)	28 (3.0%)	4.89 [2.99, 8.00]	<.001
Unmarried/widower	523 (5.7%)	457 (5.5%)	66 (7.2%)	1.92 [1.42, 2.58]	<.001
Exercise routinely					
Yes	4,062 (44.3%)	3,780 (45.8%)	282 (30.7%)	1.0	
No	5,102 (55.7%)	4,466 (54.2%)	636 (69.3%)	1.70 [1.44, 2.00]	<.001

*Note*. AMS = Aging Male Symptoms scale; OR = odds ratio.

### Association Between the Study Variables and AMS Score

Univariate analysis revealed age >40 years (OR 5.14; 95% CI [3.72, 7.10]; *p* < .001) to be highly associated with the degree of severity of AMS (AMS ≥50). In contrast, a secondary school education (OR 0.57; 95% CI [0.47, 0.69]) or postsecondary education (OR 0.30; 95% CI [0.24, 0.37]; *p* < .001) was significantly protective, compared with a primary school education (OR 1; *p* < .001). Similarly, having a monthly family income of ¥4,000–¥5,999 (OR 0.55; 95% CI [0.45, 0.68]; *p* < .001]) or ≥¥6,000 (OR 0.46; 95% CI [0.34, 0.62]; *p* < .001) was inversely associated with AMS severity. The anatomical variable WHR ≥0.9 (OR 1.33; 95% CI [1.10, 1.62];*p* < .001) was moderately associated with AMS severity. Among the lifestyle variables, smoking ≥25 cigarettes/day (OR 2.95; 95% CI [2.35, 3.69]; *p* < .001), alcohol consumption (OR 1.74 95% CI [1.50, 2.04]; *p* < .001), not exercising routinely (OR 1.70; 95% CI [1.44, 2.00]; *p* < .001), and below average marital relations (OR 1.73; 95% CI [1.47, 2.03]; *p* < .001) were moderately associated with the severity of AMS. Poor marital relations (OR 4.89; 95% CI [2.99, 8.00]; *p* < .001) were highly associated with the severity of AMS. It is noteworthy that AMS scores increased linearly with age (*p* ≤ .001).

### Univariate Analysis of Clinical Variables

As shown in Table 2, all of the clinical variables analyzed were significantly associated with the severity of AMS (*p* ≤ .05). Hypertension (OR 1.66; 95% CI [1.42, 1.94];*p* < .001), T2DM (OR 2.05; 95% CI [1.70, 2.46];*p* < .001), and a history of fracture (OR 2.78; 95% CI [1.70, 4.56]; *p* < .001) were moderately associated with AMS severity.

**Table 2.** Univariate Analysis of the Clinical Variables.

Variable	Total men (*N* = 9,164)	AMS <50 (*n* = 8,246)	AMS ≥50 (*n* = 918)	OR [95% CI]	*p* value
Hypertension					
No	6,836 (74.6)	6,285 (76.2)	551 (60.0)	1.0	
Yes	2,328 (25.4)	1,961 (23.8)	367 (40.0)	1.66 [1.42, 1.94]	<.001
Type 2 diabetes mellitus					
No	8,154 (89.0)	7,453 (90.4)	701 (76.4)	1.0	
Yes	1,010 (11.0)	793 (9.6)	217 (23.6)	2.05 [1.70, 2.46]	<.001
Prostate disease					
No	8,244 (90.0)	7,437 (90.2)	807 (87.9)	1.0	
Yes	920 (10.0)	809 (9.8)	111 (12.1)	1.16 [0.91, 1.46]	.227
Stroke history					
No	9,096 (99.3)	8,190 (99.3)	906 (98.7)	1.0	
Yes	68 (0.7)	56 (0.7)	12 (1.3)	1.93 [1.00, 3.75]	.052
Fracture history					
No	9,065 (98.9)	8,171 (99.1)	894 (97.4)	1.0	
Yes	99 (1.1)	75 (0.9)	24 (2.6)	2.78 [1.70, 4.56]	<.001

*Note*. AMS = Aging Male Symptoms scale; OR = odds ratio.

Age-stratified univariate analysis (Table 3) revealed a history of stroke (OR 12.33; 95% CI [2.34, 64.98]; *p* = .003) to be highly associated with AMS severity in men aged ≤40 years. In men aged >40 years, hypertension (OR 1.42; 95% CI [1.21, 1.66]; *p* < .001), T2DM (OR 1.86; 95% CI [1.54, 2.25]; *p* < .001), and a history of fracture (OR 2.71; 95% CI [1.59, -4.61]; *p* = .000) were moderately associated with the severity of AMS. Hypertension (*p* < .001), T2DM (*p* < .001), and benign prostate disease (*p* = .004) were correlated with AMS severity, regardless of the age of subjects.

**Table 3.** Age-Stratified Univariate Analysis of Clinical Variables and Their Association With AMS Severity.

	≤40 years (*n* = 2,135)				>40 years (*n* = 6,153)				AMS severity^a^
	AMS <50 (*n* = 2,093)	AMS ≥50 (*n* = 42)	OR [95% CI]	*p*	AMS <50 (*n* = 6,153)	AMS ≥50 (*n* = 876)	OR [95% CI]	*p*	*p*
Hypertension									
No	1,930 (92.2)	37 (88.1)	1.0	.696	4,355 (70.8)	514 (58.7)	1.0	<.001	<.001
Yes	163 (7.8)	5 (11.9)	1.21 [0.46, 3.20]		1,798 (29.2)	362 (41.3)	1.42 [1.21, 1.66]		
Diabetes mellitus									
No	2,029 (96.9)	40 (95.2)	1.0	.894	5,424 (88.1)	661 (75.5)	1.0	<.001	<.001
Yes	64 (3.1)	2 (4.8)	1.11 [0.25, 4.80]		729 (11.9)	215 (24.5)	1.86 [1.54, 2.25]		
Prostate disease									
No	1,973 (92.5)	33 (78.6)	1.0	.063	5,500 (89.4)	774 (88.4)	1.0	.399	.004
Yes	156 (7.5)	9 (21.4)	2.18 [0.96, 4.97]		653 (10.6)	102 (11.6)	1.11 [0.87, 1.42]		
Stroke									
No	2,084 (99.6)	40 (95.2)	1.0	.003	6,106 (99.2)	866 (98.9)	1.0	.253	.960
Yes	9 (0.4)	2 (4.8)	12.33 [2.34, 64.98]		47 (0.8)	10 (1.1)	1.53 [0.74, 3.16]		
Fracture									
No	2,076 (99.2)	40 (95.2)	1.0	.057	6,095 (99.1)	854 (97.5)	1.0	<.001	.121
Yes	17 (0.8)	2 (4.8)	4.43 [0.95, 20.58]		58 (0.9)	22 (2.5)	2.71 [1.59, 4.61]		

*Note*. AMS = Aging Male Symptoms scale; OR = odds ratio.

a Prevalence of clinical variable in AMS >50 group adjusted only for age.

### Multivariable Analysis of Factors Associated With the Severity of AMS

Multivariable analysis substantiated the results of univariate analysis with a marginal decrease in OR for the study variables. As shown in Table 4, age >40 years (OR 3.59; 95% CI [2.58, 5.02]; *p* < .000) and a poor marital relationship (vs. average; OR 3.58; 95% CI [2.12, 6.03]; *p* < .000) were still highly associated with AMS severity. Fracture history, which was moderately associated with univariate analysis, was found to be highly associated with AMS severity (OR 3.06; 95% CI [1.81, 5.17];*p* < .000) after adjusting for confounding variables.

**Table 4.** Multivariate Analysis of the Study Variables and Association With AMS Severity.

Factors	Adjusted OR [95% CI]	*p* value
Age >40 years	3.59 [2.58, 5.02]	<.001
Waist-to-hip ratio ≥0.9	1.40 [1.14, 1.72]	.001
<25 cigarettes per day (vs. nonsmoker)	0.93 [0.76, 1.13]	.456
≥25 cigarettes per day (vs. nonsmoker)	1.68 [1.31, 2.17]	<.001
Marital relations below average (vs. average)	1.45 [1.23, 1.72]	<.001
Marital relations poor (vs. average)	3.58 [2.12, 6.03]	<.001
Unmarried/widower (vs. average)	1.84 [1.34, 2.53]	<.001
Secondary school (vs. primary school)	0.69 [0.56, 0.84]	<.001
Postsecondary school (vs. primary school)	0.52 [0.40, 0.67]	<.001
4,000–5,999 ¥/month (vs. <4,000 ¥/month)	0.67 [0.55, 0.83]	<.001
≥6,000 ¥/month (vs. <4,000 ¥/month)	0.61 [0.44, 0.84]	.003
Alcohol consumption	1.5 [1.25, 1.80]	<.001
No exercise	1.42 [1.20, 1.70]	<.001
Hypertension	1.36 [1.16, 1.60]	<.001
Type 2 diabetes mellitus	1.84 [1.52, 2.23]	<.001
Fracture history	3.06 [1.81, 5.17]	<.001

*Note*. OR = odds ratio.

Other moderately associated factors included WHR (OR 1.40; 95 % CI [1.14, 1.72]; *p* = .001), ≥25 cigarettes per day (OR 1.68; 95% CI [1.31, 2.17]; *p* < .000), below average marital relations (OR 1.45; 95% CI [1.23, 1.72]; *p* < .000), alcohol consumption (OR 1.5; 95% CI [1.25, 1.80]; *p* < .000), lack of exercise (OR 1.42; 95% CI [1.20, 1.70]; *p* < .000), hypertension (OR 1.36; 95% CI [1.16, 1.60]; *p* < .000), and T2DM (OR 1.84; 95% CI [1.52, 2.23]; *p* < .000).

## Discussion

The present study found that age, WHR, smoking, alcohol consumption, marital relationship, and physical exercise influence the quality of life in the aging male according to the AMS scale. It was revealed that 10% of the overall study population had severe AMS (AMS >50), with the incidence in men >40 years being 12% and in men ≤40 years being 2%. The prevalence of a severe AMS score in men <40 years of age was 2% in the current study, which is close to the corresponding prevalence reported in France (1.5%) (Myon, Martin, Taïeb, & Heinemann, 2005). Four previous Chinese studies utilized the AMS questionnaire in a relatively small population of men recruited within urban areas of China (W. Chen et al., 2013; Chueh et al., 2012; Li et al., 2016; Sun et al., 2012) and only one of the studies reported AMS ≥50, which was 8.2% (Li et al., 2016); this was consistent with our analysis in a much larger cross-sectional study. An international Web survey that included mainly participants from the United Kingdom and the United States found a prevalence as high as 49.7% of AMS ≥50 peaking in men in their 50s. However, the authors noted that the survey was biased toward participants who were searching the Web for an explanation of symptoms, which might be related to testosterone deficiency, and 26% of them gave a positive response to the additional question of whether they underwent vasectomy (Trinick, Feneley, Welford, & Carruthers, 2011).

Among the demographic variables analyzed, age, education, and monthly income were associated with the degree of severity of AMS. A positive association between age and severity of AMS has also been reported in previous studies (Ichioka et al., 2006; Myon et al., 2005).

Secondary and postsecondary school education together with a higher family income was found to be negatively associated or protective in the present study, which is in agreement with previous research (Jankowska et al., 2008)

Among the anatomical and lifestyle variables, WHR, smoking, alcohol consumption, and marital relationship were mildly to highly associated with the severity of AMS. The association of WHR, which is a better indicator of obesity than BMI in the elderly, substantiates the usefulness of WHR as the optimum anthropometric in the elderly (Srikanthan, Seeman, & Karlamangla, 2009). The positive association between smoking and the degree of severity of AMS may be indirectly attributed to its association with hypertension.

Poor marital relationship was a predictor for AMS severity in the present study. Since the marital relationship is strongly influenced by the frequency of sexual intercourse, which has been shown to decrease with both increasing age and longer marriage duration (Wylie & Kenney, 2010), it is difficult to predict whether a poor marital relationship leads to severe AMS or vice versa. Being unmarried or a widower, although moderately associated, may contribute to AMS severity by increasing depression due to lack of companionship.

Among the clinical variables, hypertension, T2DM, and a history of fracture clearly influenced the severity of AMS. Because T2DM is a comorbidity for androgen deficiency (Corona et al., 2011), it was associated with AMS severity in our study. History of fracture was also included as a clinical variable since it is indicative of the cumulative fall risk in aging men, which has been reported to increase with advancing age (Saad, Röhrig, von Haehling, & Traish, 2017).

Although the current study was cross-sectional in nature, some of the study variables could be considered as risk factors since they were not subject to temporal changes. From this perspective, age, income, education, and smoking are prospective risk factors for AMS severity. Further prospective longitudinal studies may validate the preliminary claim of this study.

The major limitation of the study is its cross-sectional nature in which the factors favoring progression of AMS severity could not be ascertained. No analysis was carried out to compare the factors associated with urban and rural samples. Furthermore, attitudes toward sex-related questions varied between cultures.

## Conclusions

We performed a nationally representative epidemiological survey of AMS scores for 9,164 Chinese men aged between 35 and 70 years old. Data were collected from 33 community health centers at locations in all regions of China, with the exception of Hong Kong, Taiwan, Macau, Tibet, and Qinghai. The overall prevalence of severe AMS scores was 10.0% and the AMS scores increased linearly with age. Age-stratified univariate analysis identified hypertension and T2DM as major contributors to the severity of AMS. Further clinical studies will be required to investigate how these risk factors interact to have an impact on HRQoL in middle-aged and elderly men.
